# Anti-inflammatory Mechanism of Geniposide: Inhibiting the Hyperpermeability of Fibroblast-Like Synoviocytes via the RhoA/p38MAPK/NF-κB/F-Actin Signal Pathway

**DOI:** 10.3389/fphar.2018.00105

**Published:** 2018-02-15

**Authors:** Ran Deng, Feng Li, Hong Wu, Wen-yu Wang, Li Dai, Zheng-rong Zhang, Jun Fu

**Affiliations:** ^1^Key Laboratory of Xin’an Medicine, Ministry of Education, Hefei, China; ^2^College of Pharmacy, Anhui University of Chinese Medicine, Hefei, China

## Abstract

Geniposide (GE) is the extraction and purification of iridoid glycosides from the *Gardenia jasminoides Ellis*, which is a promising anti-inflammatory drug, but its mechanism of actions on rheumatoid arthritis (RA) has not been clarified. This study investigated the molecular mechanism behind GE reduced the high permeability of fibroblast-like synoviocytes (FLSs) derived from SD rats with adjuvant arthritis (AA), with the aims of observing the action of GE in AA rats and exploring new therapeutic strategies for RA treatment. The CCK-8 method was used to detect FLSs proliferation. The pro-inflammatory cytokines levels and anti-inflammatory cytokines levels in FLSs were determined by ELISA kits. FLSs permeability assay was performed on Transwell. Immunofluorescence was used to assay the arrangement and morphology of F-actin. The expression of the key molecules related to FLSs permeability (RhoA, p-p38MAPK, NF-κB p-p65 and F-actin) was detected by western blotting. After treatment with lipopolysaccharide (LPS), the proliferation and the permeability of the cells increased significantly (all *P* < 0.05). The expression of RhoA, p-p38MAPK, NF-κB p-p65 and F-actin in FLSs was higher compared with the control group, and F-actin was redistributed, with the formation of additional stress fibers. But, these conditions were moderated after treatment with GE. We demonstrated that the treatment of different concentrations of GE (25, 50, and 100 μg/mL) had a significant inhibitory effect on the proliferation and permeability of FLSs *in vitro*. Furthermore, the levels of interleukin (IL)-1β and IL-17 secreted by FLSs were decreased in different doses of GE groups, and the levels of anti-inflammatory cytokines (IL-4, TGF-β1) were increased. Under treatment with GE, low expression of RhoA downregulated expression of p-p38MAPK, NF-κB p-p65, and F-actin while compared with control group, and restored the hyperpermeability of FLSs due to LPS treatment. Taken together, GE might play its anti-inflammatory and immunoregulatory effects via regulating the relative equilibrium of pro-inflammatory cytokines and anti-inflammatory cytokines. GE attenuated the hyperpermeability of FLSs. The down-regulation of the conduction of RhoA/p38MAPK/NF-κB/F-actin signal may play a critical role in the mechanisms of GE on RA. GE could be an effective therapeutic agent for the treatment of RA.

## Introduction

Rheumatoid arthritis (RA) is a systemic, progressive and chronic inflammatory immune disease (Firestein, 2003; Firestein and McInnes, 2017), which is characterized by synovial tissue inflammation, the formation of synovial pannus, and progressive joint damage of cartilage and bone (See et al., 2013; Mankia and Emery, 2015; Chang et al., 2015). Abnormal proliferation of fibroblast-like synoviocytes (FLSs) is a central event in the development of RA (Filer, 2013). FLSs show tumor cell-like growth. It can secrete large amounts of inflammatory cytokines, chemokines, matrix metalloproteinases (MMPs), regulate the formation of pannus and destruct cartilage and bone, eventually lead to joint deformity and disfunction. It is reported that pro-inflammatory cytokines are activated by multiple signaling pathways, such as the RhoA/ROCK, mitogen-activated protein kinases (MAPKs) and nuclear factor κB (NF-κB) pathways (Xiao et al., 2013; Zhuang et al., 2017). Recent studies found that MAPKs, NF-κB, and Rho/ROCK pathways are highly activated in FLSs and involved in the pathogenesis of RA (Ganesan et al., 2016; Liu et al., 2017; Yokota et al., 2017). Blocking MAPKs, NF-κB and Rho/ROCK signaling pathways to find a new anti- inflammatory drugs for RA treatment has been a focus.

Geniposide (GE), a kind of iridoid glycosides, derived from *Gardenia jasminoides Ellis*, with anti-inflammatory, anti-oxidation and anti-angiogenesis and other pharmacological effects (Fu et al., 2012; Xiaofeng et al., 2012). Our previous studies found that GE could control the activity of cytokines and decrease the levels of inflammatory medias in adjuvant arthritis (AA) rats (Wu et al., 2008). It can inhibit the proliferation of FLSs and decrease the levels of Interferon-gamma (IFN-γ), IL-6, IL-17 and other inflammatory cytokines levels and increase the level of anti-inflammatory cytokines such as TGF-β1, IL-4 that secreted by FLSs or mesenteric lymph node (Dai et al., 2014; Wang R. et al., 2017). These effects were related to the amelioration of Jun N-terminal kinase (JNK) signaling and p38 signaling of MAPKs signaling cascade in FLSs (Chen J. et al., 2015). All of these studies indicated that GE may play a key role in regulating FLSs permeability. MAPKs signaling pathways are important for extracellular signals that cause fine internal reactions. In RA tissue, p38MAPK is activated in synovial lining and endothelial cells. It was found that p38MAPK was activated by inflammatory stimulant (LPS, IL-1, IL-6, etc.), mitogen, growth factor and so on. Activated p38MAPK could regulate the expressions of MMPs, NF-κB and fibrinous actin (F-actin), etc., which played an important role in the pathogenesis of RA joint swelling, synoviocyte dysplasia, angiogenesis, articular cartilage and bone erosion and progressive destruction (Li et al., 2013; Criado et al., 2014; Wang et al., 2015; Zou et al., 2016).

Cytoskeleton is a complex fibrous network composed of protein filaments in the cytoplasm. It mainly consists of three kinds of protein fibers: microtubules, microfilaments and intermediate fibers (Singer et al., 1985). Cytoskeleton plays an important role in maintaining cell morphology, such as cell survival, migration, apoptosis and the transport of substances inside and outside the membrane. Actin is a structural protein microfilament, mainly in both monomer and polymer form. Monomeric actin, also known as globular actin (G-actin) is composed of a polypeptide chain spherical molecules. F-actin filaments are actin filaments formed by actin multimers and are cytoskeletal fibers with a diameter of about 7 nm (Puthenedam et al., 2007). The actin cytoskeletal network plays a major role in the maintenance of the endothelial barrier function, as it regulates the integrity of the cells. In inflammatory cytokines, growth factors, poisoning, hypoxia, ischemia and other pathological conditions, those can cause F-actin intracellular morphological and distribution changes, lead to some physiological barrier permeability increased, thus impairing its function (Connolly et al., 2011). Ras superfamily related RhoA/Rho pathway exists in many organisms. The regulation of intracellular actin polymerization and depolymerization by the RhoA/Rho pathway is responsible for the “molecular switch” that in turn triggers the downstream kinase cascade to play a biological role. RhoA/ROCK pathway is an important regulator of cell morphology and F-actin. It cannot only maintain the cell morphology, but also affect the cell adhesion through the correlation with the extracellular matrix and extracellular matrix. It plays a crucial role in various physiological activities such as cell migration, growth, proliferation and apoptosis (Dasgupta et al., 2017; Wei et al., 2017).

The mechanism of FLSs hyperpermeability induced by inflammatory cytokines has been illuminated to a certain extent. RhoA/ROCK signaling pathway plays a key role in the regulation of different cells permeability and barrier function (Wang et al., 2012; Yin et al., 2016; Dong et al., 2017). The study found that F-actin was disorder in bone marrow mesenchymal stem cells (MSC) in patients with systemic lupus erythematosus (SLE) (Dong et al., 2015). Protein expression was significantly higher than the normal control group; the expression levels of RhoA and F-actin were overexpressed in the MH7A cell line induced by IL-1β (10 ng/mL) in RA patients (Lv et al., 2015). The increase of the F-actin expression in many diseases has been confirmed (Puthenedam et al., 2007; Ng et al., 2015). Kawanami et al. (2011) found that ROCK-specific inhibitor Y-27632 inhibited the phosphorylation of p38MAPK and p65, which indicated that ROCK was able to regulate p38MAPK/NF-κB signaling pathway. Thus, the intervention of F-actin by RhoA through p38MAPK/NF-κB signaling pathway may be a new target for RA therapy.

In this study, FLSs isolated from AA rats have been used to determine the effectiveness of GE. Furthermore, we used LPS-treated FLSs, observed the influence of GE on FLSs hyperpermeability and assessed the effect of GE on the RhoA/p38MAP/NF-κB/F-actin signaling pathway and then constructed a new therapeutic effect target.

## Materials and Methods

### Materials and Reagents

Geniposide was extracted and purified from *G. jasminoides* Ellis (purity > 98%), and provided by the National Institute for the Control of Pharmaceutical and Biological Products (Beijing, China). Fetal bovine serum (FBS) was obtained from HyClone (Logan, United States). Rabbit monoclonal antibodies against p38, p-p38, NF-κB p65 and NF-κB p65 were supplied from Cell Signaling Technology (Beverly, MA, United States). Anti-RhoA antibody was purchased from Abcam (Cambridge, MA, United States). β-actin and HRP-conjugated goat anti-rabbit antibodies were provided by ZSGB-BIO (Beijing, China). Rho-kinase inhibitor (Y-27632) was obtained from Selleck (Houston, TX, United States). Rat IL-4, IL-1β, IL-17 and TGF-β ELISA kits were purchased from Elabscience Biotechnology (Wuhan, China). Freund’s Complete Adjuvant (FCA) and LPS were supplied from Sigma Chemical Company (St. Louis, MO, United States). Other chemicals that have been used in this work were of research grade.

### Animals

Male Sprague-Dawley (SD) rats (200 ± 20 g, Grade II, Certificate NO.078) were purchased from the Experimental Animal Center of Anhui University of Chinese Medicine (Hefei, China). All rats were housed under specific pathogen-free conditions with a 12-h light/dark cycle in a temperature-controlled room at 25 ± 1°C and 50–60% relative humidity. All rats were maintained in this condition at least 7 days before experiment. All studies on rats were carried out following the Guideline for Animal Experiments of Anhui University of Chinese Medicine and the protocol was approved by the Ethics Review Committee for Animal Experimentation of Anhui University of Chinese Medicine.

### Induction and Evaluation of Adjuvant Arthritis Rats

Adjuvant arthritis models were induced according to the previous method. Rats were immunized on day 0 by a single intradermal injection into the right hind paw with 100 μL of FCA, while the normal group rats were given the same volume of physiological saline at the same time (*n* = 12). Rats were randomly divided into two groups: normal control group, AA model group, 6 rats in each group. At the day 7, day 11, day 14, day 17, day 21, two groups of rats were evaluated from the paw volume, arthritis index and arthritis systemic assessment. All the rats were examined for signs of arthritis by two independent and blind to the experimental design of observers. Non-injected hind paws volume of rats were measured with PV-200 volume meter (Chengdu Taimeng Technology Co., Ltd., Chengdu, China). The degree of secondary joint swelling in rats was calculated as (ΔmL = volume of the foot after modeling – the volume of the foot before modeling).

The arthritis systemic assessment was scored according to the degree of swelling of the fore foot and posterior foot of the rat and the presence of nodules and erythema in the nasal, ear and tail. The specific standard is as follows: Nose: 0 = no connective tissue swelling, 1 = significant connective tissue swelling; Ears: 0 = No nodules and redness, 1 = nodules and redness appear in one ear, 2 = nodules and redness appear in both ears; Tail: 0 = no nodules, 1 = nodules; Forefoot: 0 = no swelling, 1 = paw swelling in one forefoot, 2 = paw swelling in both forepaws; Hind paw: 0 = no swelling, 1 = swelling of one hind paw, 2 = swelling of both hindpaws. Each rat scored up to 8 points.

The arthritic index was based on the occurrence and severity of secondary lesions of arthritis in rats. Grading standards are evaluated as follows: 0 = no redness; 1 = redness of the foot metatarsal joint; 2 = redness of the toe joint and toes; 3 = redness below the ankle joint; 4 = redness of the entire foot, including the ankle joint. Each rat has a maximum of 12 points.

### Preparation and Culture of FLSs

**FIGURE 1:**
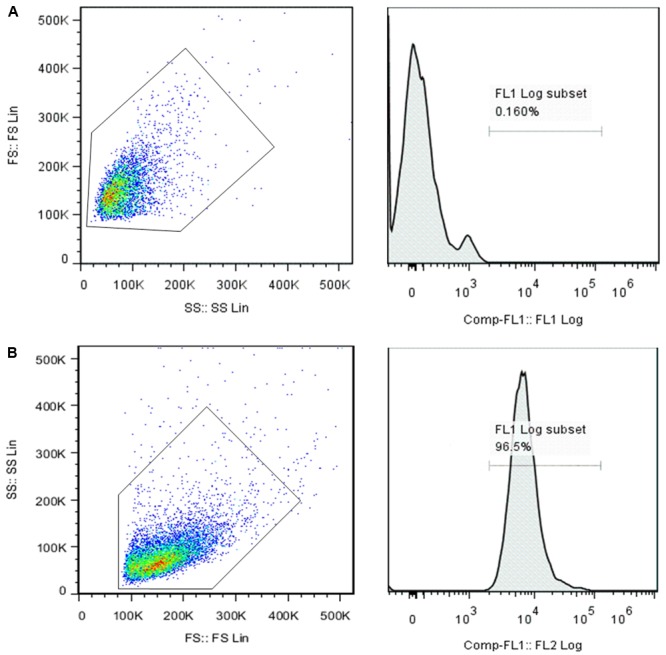
Flow cytometry analysis of the surface maker of the FLSs. Mouse IgG isotypes labeled with FITC served as a negative control **(A)**. The positive rate of 0.16% was chosen as the background (as shown in the box) and then the FITC-labeled VCAM-1 **(B)** expression rate was examined. The results showed that the expression of VCAM-1 in the cell suspension was 99.5%, indicating that the main component of cultured third-generation synoviocytes was FLSs.

Fibroblast-like synoviocytes from AA rats synovial tissues were isolated by the method of tissue explant cultivation as described previously (Wei et al., 1986). AA rats were anesthetized and sacrificed by bleeding from the abdominal aorta on day 21 after immunization. The fresh synovial tissue were taken out in sterile condition and put into culture dishes with D-Hank’s solution, washed and removed of all fat and connective tissue. Then, AA rats synovial tissues were cut into small pieces of 1–2 mm^3^, incubated in a flat bottom culture bottle and cultured in DMEM supplemented with 20% FBS at 37°C and 5% CO_2_. The culture solution was changed every 2–3 days. When a large number of FLSs grew from the synovial tissue, small pieces of tissue were discarded. Adherent cells were trypsinized, cells were routinely split at a 1:2/1:3 ratio, and cultured in medium. According to the growth of FLSs and the change of the color of the culture medium, the fluid was changed once every 1–2 days. The FLSs obtained from the 3th to 4th passages were used for additional experiments. Flow cytometry analysis was used to identify the purity of FLSs according to the expression of vascular cell adhesion molecule-1 (VCAM-1). The results indicated that the expression of VCAM-1 in passaged third synovial cells was 99.5% (**Figure 1**). The results showed that the third generation of cell suspension cultured mainly FLSs.

### Proliferation Assay by CCK-8

Fibroblast-like synoviocytes obtained from AA rats were planted into 96-well plate at a concentration of 1 × 10^5^ cells/mL containing 100 μL DMEM medium with 10% FBS at 37°C for 24 h in a 5% CO_2_ incubator. The FLSs were divided into control group, AA group, different doses of GE groups (6.25, 12.5, 25, 50, and 100 μg/mL) and Y-27632 inhibitor group. The cells were incubated at 37°C for 48 h in a 5% CO_2_ incubator. Subsequently, the cells were washed with PBS twice. Then, 10 μL CCK-8 (Japanese colleagues Chemical, Japan) was added to each well, and cells were further incubated for another 4 h. The optical density values of each well were examined at 450 nm using a microplate reader (Haiou Pei Analytical Instruments Co., Ltd., China, Shanghai).

### FLSs Monolayer Permeability/Barrier Function

Bovine serum albumin (BSA) diffusion through FLSs monolayers was evaluated using transwell cell culture chambers (collagen-coated polycarbonate filters with a pore size of 0.4 μm). A cell suspension (1 × 10^5^ cells) was added to the upper chambers of the Transwell apparatus. FLSs seeded in the upper chambers of transwells were maintained in culture for a period of 12 days to obtain monolayers, with culture media being changed on alternate days. To measure monolayer barrier function, cell culture medium was replaced with media containing BSA (200 mg/L), before initiating drug treatment for 0.5 h at 37°C. Then, the cells were divided into six groups (*n* = 3 transwells): control group, AA group, GE groups (25, 50, and 100 μg/mL), Y-27632 (10 μM). After 48 h of drug treatment, media from the lower chamber were collected to monitor BSA content by Bradford method.

### The Induction and Activity of IL-1β, IL-17, IL-4, and TGF-β1 (Talaat et al., 2015)

The FLSs of AA rats were planted into six-well plates at a density of 2 × 10^5^ cells/well at 37°C in a 5% CO_2_ incubator. Then, the cells were divided into six groups: control group, AA group, GE groups (25, 50, and 100 μg/mL), Y-27632 (10 μM). The supernatants were collected to measure the levels of IL-1β, IL-17, IL-4, and TGF-β1. The cytokine levels in the culture supernatants of FLSs were determined by using murine or rat specific ELISA kits for IL-1β, IL-17, IL-4, and TGF-β1 according to the manufacturer’s instructions (Elabscience Biotechnology, Wuhan, China).

### Immunofluorescence Staining

To further observe changes of F-actin in FLSs, immunofluorescent staining was performed. FLSs were detached with 0.25% Trypsin-EDTA. Cultured FLSs under different conditions were plated onto different 24-well plates and fixed in 4% paraformaldehyde for 10 min, followed by 1% BSA (with 0.3% Triton X-100) for 1 h at room temperature. The F-actin of FLSs were incubated with 200 μL FITC-conjugated phalloidin (150 nM) (Sigma). DAPI was used to visualize the nuclei at room temperature for 1 h. Samples were examined using a confocal laser scanning microscope.

### Western Blotting Analysis

The standard method was used to analysis of the proteins extracted from FLSs by Western blot. The protein concentration was assayed by BCA Protein Assay Kit. The proteins were separated by 10% sodium dodecyl-sulfate-polyacrylamide gel electrophoresis (SDS-PAGE) and were electrophoretically transferred to nitrocellulose (NC) membranes (Millipore, Bedford, MA, United States). After blockage with 5% skim milk at room temperature for 2 h, the membranes were incubated with the specific primary antibodies against β-actin, RhoA, p38, p-p38, p65, p-p65, and F-actin at 4°C overnight on a rotary shaker. The membranes were washed in Tris-buffered saline/Tween 20 (TBST) and then incubated horse radish peroxidase conjugated secondary antibody for 2 h. The membranes were washed in TBST again and the immunoreactive proteins were detected with Pierce^TM^ ECL Western Blotting Substrate (Thermo Scientific, MA, United States). The protein bands were scanned by Alpha View SA system (ProteinSimple, San Jose, CA, United States) and analyzed using Image software.

### Statistical Analysis

All data were expressed as mean ± standard deviation (SD). The Statistical analysis was performed by SPSS 21.0 software, and the differences between groups were carried out by Student’s *t*-test and One-way ANOVA test. Value of *P* < 0.05 or *P* < 0.01 was considered statistically significant.

## Results

### Evaluation of Arthritis on AA Rats

**FIGURE 2:**
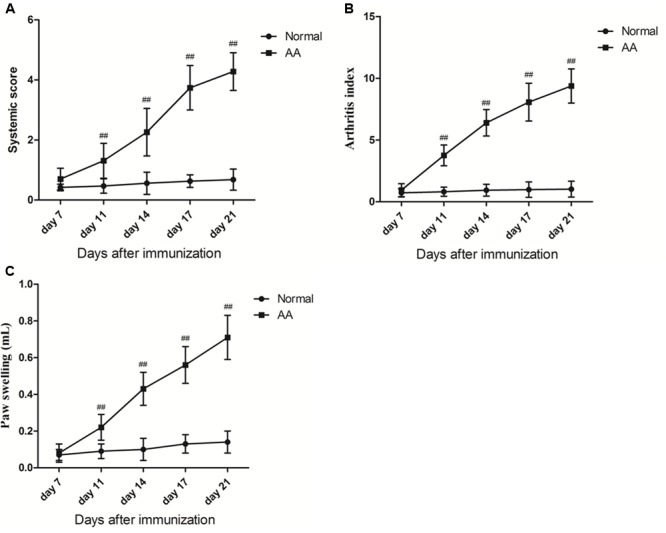
**(A)** The systemic score assessment of normal group and AA group rats (*x* ±*s, n* = 6). **(B)** The inflammatory poly arthritis index score of normal group and AA group rats (*x* ±*s, n* = 6). **(C)** Secondary normal group and AA group rats foot swelling of change (ΔmL) (*x* ±*s, n* = 6). Rats were immunized on day 0 by a single intradermal injection into the right hind paw with 100 μL of FCA. At the day 7, day 11, day 14, day 17, day 21, two groups of rats were evaluated from the paw volume, arthritis index and arthritis systemic assessment. Data are presented as means ± SD. ^##^*P* < 0.01 vs. normal group.

To extract FLSs primary cells from AA rats, the AA models in SD mice were employed. After healthy SD rats were immunized with FCA for the first time, d1–d3 showed redness in the right side of the rat, which was caused by subcutaneous injection of inflammation. On the 7th day after immunization, the primary swelling of right hind paws in AA rats was reappeared. On day 11 after immunization, the secondary swelling of left hind paws in AA rats arose obvious secondary inflammatory response, which showed paw swelling, pain, polyarthritis, nose, ear and tail appeared nodules and erythema. The systemic score assessment, polyarthritis index, and right hind paw swelling of AA rats were significantly increased compared with normal rats (*P* < 0.01). The results were shown in **Figure 2**. In this study, typical and stable AA rats were successfully established.

### GE Inhibited the Proliferation of FLSs in AA Rats

**FIGURE 3:**
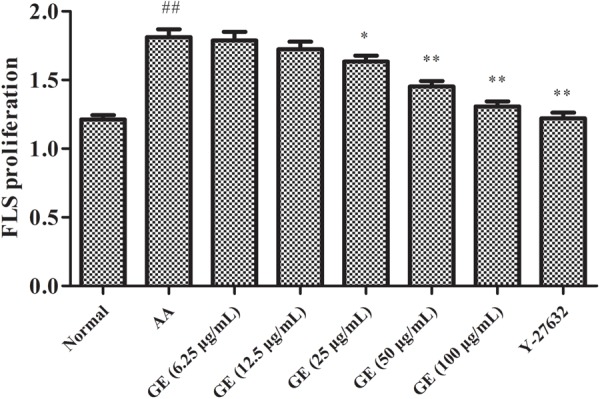
Effects of different concentrations of GE on the proliferation of FLSs on AA rats *in vitro* (*n* = 3). Data were obtained from three independent experiments and are expressed as the mean ± SD. ^##^*P* < 0.01 vs. control group; ^∗^*P* < 0.05, ^∗∗^*P* < 0.01 vs. AA group.

As shown in **Figure 3**, the study results of CCK-8 suggested that the optimal concentration of GE were 25, 50, and 100 μg/mL for FLSs proliferation. Thus, 25, 50, and 100 μg/mL were chosen as the appropriate concentrations of GE in the next test. The proliferation activity of FLSs in AA group enhanced significantly compared to control group (*P* < 0.01). Compared with AA model group, GE groups (25, 50, and 100 μg/mL) had a significant inhibitory effect on LPS-induced FLSs cell proliferation (*P* < 0.01), Y-27632 inhibitor group (10 μM) also showed significantly inhibitory on proliferation of FLSs which maybe related RohA/ROCK pathway (*P* < 0.01).

### GE Relieved LPS-Induced Hyperpermeability of FLSs

**FIGURE 4:**
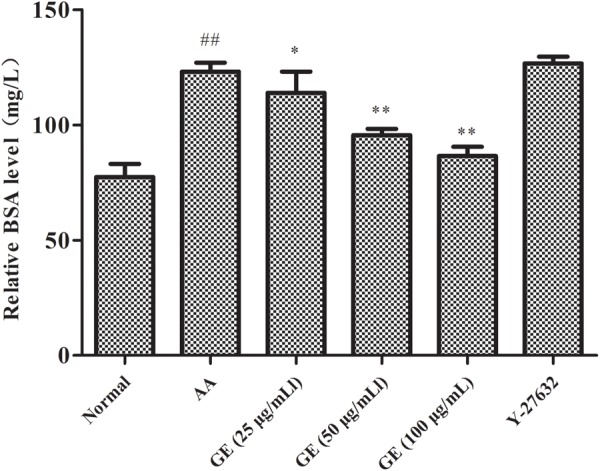
Effects of GE on monolayer permeability/barrier function in FLSs (*n* = 3). Data were obtained from three independent experiments and are expressed as the mean ± SD. ^##^*P* < 0.01 vs. control group; ^∗^*P* < 0.05, ^∗∗^*P* < 0.01 vs. AA group.

Using monolayers of FLSs in a Transwell apparatus, we tested whether GE can directly affect the high permeability of FLSs induced by LPS via evaluating the permeability to BSA. As seen in **Figure 4**, the results showed that LPS significantly increased the permeability of FLSs, indicating the formation of hyperpermeability of FLSs. In GE groups (25, 50, and 100 μg/mL) (*n* = 3), transport of BSA across the FLSs was decreased following the addition of GE compared to the untreated LPS group and exhibited a dose-dependent manner. And FLSs permeability was positively correlated with the relative concentration of BSA in the lower chambers of transwell medium of each group.

### Effects of GE on Cytokines Secretion of FLSs

**FIGURE 5:**
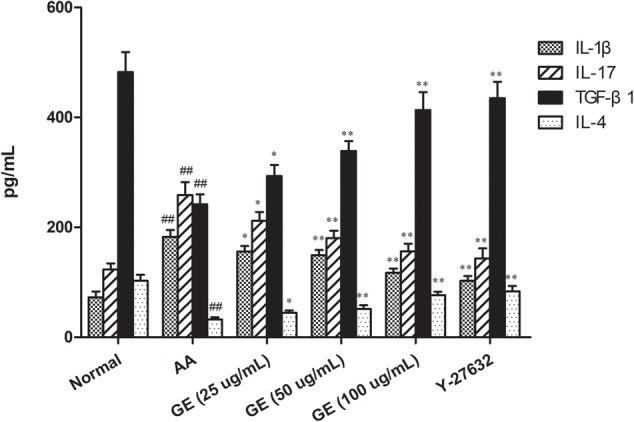
Effects of GE on production of IL-1β, IL-17, TGF-β1, and IL-4 in FLSs of AA rats. The cell supernatants were collected to detect the levels of IL-1, IL-17, TGF-β1, and IL-4 by ELISA (*n* = 3). Data were obtained from three independent experiments and are expressed as the mean ± SD. ^##^*P* < 0.01 vs. control group; ^∗^*P* < 0.05, ^∗∗^*P* < 0.01 vs. AA group.

To confirm the anti-inflammatory effects *in vitro*, GE was evaluated for its inhibition of pro-inflammatory (IL-1β and IL-17) mediators and increase of anti-inflammatory (IL-4 and TGF-β1) in FLSs stimulated by LPS. The result was shown in **Figure 5**. The results of ELISA showed that the levels of IL-1β and IL-17 in FLSs were significantly up-regulated compared with the normal group, and the levels of anti-inflammatory cytokines IL-4 and TGF-β1 was significantly declined (*P* < 0.01). Compared with AA model group, different doses of GE groups (25, 50, and 100 μg/mL) could significantly decrease the secretion of IL-1β and IL-17 in FLSs, and significantly increase the secretion of IL-4 and TGF- β1 (*P* < 0.01). The levels of IL-1β and IL-17 in FLSs were significantly decreased while treatment with Y-27632 inhibitor, and the levels of IL-4 and TGF-β1 were significantly increased (*P* < 0.01).

### GE Affected the Distribution of F-Actin in FLSs Induced by LPS

**FIGURE 6:**
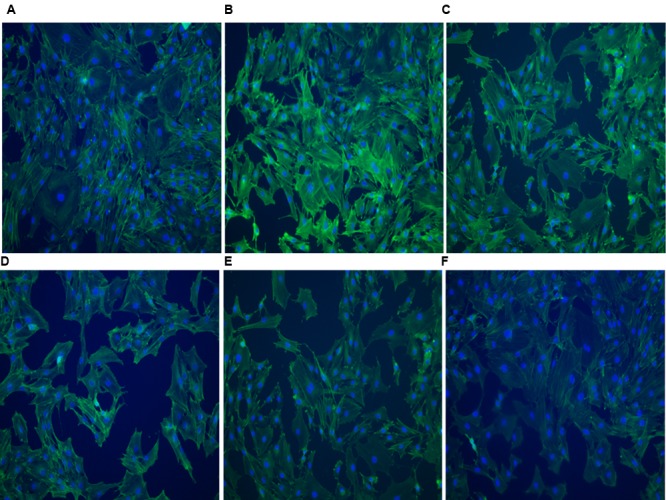
Immunofluorescence images of F-actin of FLSs in AA rats (*n* = 3) (200×). FITC-phalloidin labeled F-actin showed green fluorescence and DAPI-labeled nuclei showed blue fluorescence. **(A)** Normal group, **(B)** AA group, **(C)** GE (25 μg/mL) group, **(D)** GE (50 μg/mL) group, **(E)** GE (100 μg/mL) group, **(F)** Y-27632 inhibitor group (10 μM).

In order to observe changes in the cytoskeleton, immunofluorescence was used to observe F-actin in FLSs. It could be observed in **Figure 6** that under confocal laser scanning microscope, FITC-phalloidin labeled F-actin showed green fluorescence and DAPI-labeled nuclei showed blue fluorescence. We observed that F-actin is mainly located in the outer periphery of the cell and inside the cell membrane, with a complete cell membrane and clear cell borders. In contrast, when FLSs were incubated with LPS, cell boundaries became obscured and dense F-actin stress fibers accumulated. FLSs also induced an increase in F-actin, as determined by western blot compared with normal group. The doses of GE (25, 50, and 100 μg/mL) could reduce F-actin fibers in FLSs cytoplasm in different degrees, and F-actin fibers accumulated on the periphery of the membrane. Moreover, when FLSs pretreated with Y27632, the location and the amount of F-actin resembled that of unstimulated cells, thus indicating that this pathway may be responsible for AA-induced cytoskeletal rearrangement.

### GE Inhibited the High Expression of RhoA, p-p38MAPK, NF-κB p-p65, and F-Actin Protein of FLSs Induced by LPS

**FIGURE 7:**
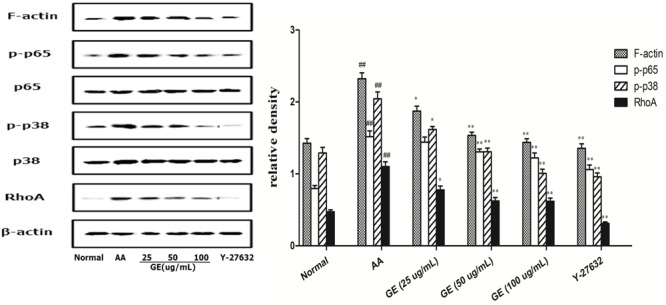
Effects of GE on expression of RhoA, p38MAPK, NF-κB and F-actin in FLSs of AA rats. Western blotting analysis was performed to detect the expression levels of RhoA, p-p38MAPK, NF-κB p-p65 and F-actin (*n* = 3). The results were expressed as means ± SD from three independent experiments. ^##^*P* < 0.01 vs. control group; ^∗^*P* < 0.05, ^∗∗^*P* < 0.01 vs. AA group. a: normal group, b: AA group, c: GE (25 μg/mL) group, d: GE (50 μg/mL) group, e: GE (100 μg/mL) group, f: Y-27632 inhibitor group (10 μM).

We have already identified AA did enhance FLSs permeability, then we wish to determine whether AA induced permeability increase via this pathway. First, in order to confirm whether AA induced activation of RhoA/p38MAPK/NF-κB/F-actin pathway in FLSs, The phosphorylation of RhoA, p-p38MAPK, NF-κB p-p65, and F-actin was identified. The experimental results of protein electrophoresis were shown in **Figure 7**. The expression of RhoA, p-p38MAPK, NF-κB p-p65, and F-actin of FLSs in AA model group was significantly upregulated while compared with normal group (*P* < 0.01). The results demonstrated that RhoA/p38MAPK/NF-κB/F-actin pathway are highly activated in FLSs by LPS. Different doses of GE groups (25, 50, and 100 μg/mL) significantly reduced RhoA, p-p38MAPK expression and F-actin protein in FLSs (*P* < 0.01). There was significant difference in the expression level of NF-κ B p-p65 protein (*P* < 0.01) only in the middle and high dose of GE groups (50 and 100 μg/mL). The levels of RhoA, p-p38MAPK, NF-κB p-p65 and F-actin proteins in FLSs was also significantly reduced by Y-27632 inhibitor group (10 μM) (*P* < 0.01).

## Discussion

Rheumatoid arthritis is a classic systemic autoimmune disease characterized by persistent chronic synovitis, vascularization, further destruction of articular cartilage and bone, and even deformity and serious complications (Walsh et al., 2010; Kong et al., 2016). Synovial cells are the major effector cells during RA. FCA induced AA model is a chronic, systemic, autoimmune inflammation mediated by T cells. It is very similar to human RA in histopathological and laboratory parameters and is ideal models for screening and studying drugs for treating RA (Chen C.X et al., 2015; Qian et al., 2017). AA rats mainly manifested as multiple peripheral arthritis and local joint swelling, deformity, pathological changes of proliferative synovitis, articular cartilage damage, bone erosion, inflammatory cell infiltration. And AA synovial also appeared neovascularization phenomenon, which is similar to human RA. Therefore, the AA rat model is an ideal model for screening and studying RA therapies.

Fibroblast-like synoviocytes play an important role in maintaining the homeostasis of the joint cavity in the normal body. In RA, a large number of harmful substances (inflammatory cytokines, inflammatory mediators, etc.) gathered in the joint cavity, abnormal proliferation and secretion of synovial cells, eventually leaded to articular cartilage and bone destruction (Byng-Maddick and Ehrenstein, 2015; Firestein and McInnes, 2017). Abnormal changes in rheumatoid arthritis synovial cells (RASFs) play a major role in the pathogenesis of RA. In the normal body, synovial cells control the structural and dynamic integrity of the joint movement by controlling the synovial fluid and the composition of the extracellular matrix in the joint lining. However, RASFs exhibit surprising pathogenic behavior, which increase in number, destroy vasoconstrictions, and have a serious effect on the induction of RA inflammation and joint damage. In this study, the proliferation of FLSs in AA rats induced by LPS was significantly faster than that in normal controls, while different concentrations of GE (25, 50, and 100 μg/mL) and Y-27632 inhibitor (10 μM) could significantly inhibit the proliferation of FLSs, suggesting that GE has an inhibitory effect on abnormal proliferation of FLSs obtained from AA rats.

Rheumatoid arthritis is a typical progressive, systemic inflammatory disease, whose major pathologic changes are associated with lymphocyte, synoviocyte synthesis of inflammatory cytokines and their receptors associated with abnormal gene expressions. The immune dysfunction is considered to be the primary pathogenesis of RA (Chakera et al., 2012; Lubberts, 2015; Tan et al., 2017). In the normal body, anti-inflammatory and pro-inflammatory cytokines maintain a dynamic balance, but this balance is broken in RA. IL-1β and IL-17 cannot only induce the proliferation of FLSs, but also stimulate the angiogenesis by promoting the expression of vascular endothelial growth factor (VEGF), IL-8, MPPs and other forms of angiogenesis, leading to the formation of pannus and synovial lesions of RA (Kim et al., 2017; Wang Y. et al., 2017). IL-4 is an important anti-inflammatory cytokines, which can enhance neutrophil-mediated phagocytosis and cytotoxicity and play an important role in maintaining humoral immunity *in vivo* (Krabben et al., 2013). TGF-β1 is an anti-inflammatory cytokine that promotes the growth of fibroblasts and osteoblasts, and the expression of extracellular matrix such as collagen and fibronectin, thereby inhibiting inflammation and autoimmune responses (Suzuki et al., 2015). The balance was skewed toward pro-inflammatory cytokines, resulting in excessive production of IL-17 and IL-1β, whereas treg activity and the level of TGF-β1 were deficient in RA patients compared to healthy individuals. In this study, the levels of IL-1β, IL-17, IL-4, and TGF-β1 were evaluated to investigate whether the imbalance of pro-inflammatory cytokines and anti-inflammatory cytokines was involved in the inflammatory injury. The levels of pro-inflammatory cytokines IL-1β and IL-17 increased significantly in AA rats, while the levels of anti-inflammatory cytokines IL-4 and TGF-β1 decreased significantly, suggesting that the inflammatory symptoms were related to the expression levels of IL-1β, IL-4, IL-17, and TGF- β1 in FLSs. The levels of IL-1β and IL-17 in FLSs were significantly inhibited while the levels of IL-4 and TGF-β1 were up-regulated in different doses of GE groups (25, 50, and 100 μg/mL). While treatment with Y- 27632 inhibitor, the effect was similar to that of GE. These results suggested that inhibition of the levels of inflammatory cytokines, upregulation of anti-inflammatory cytokines, recovery of the dynamic balance between pro-inflammatory cytokines and anti-inflammatory cytokines are an important characteristic of GE regulation of immune balance.

Cytoskeleton is the basis for maintaining cell traits and positions, including microtubules, microfilaments and intermediate fibers. Actin is a structural protein of microfilaments in the forms of G-actin and F-actin. Under various stimuli, G-actin is polymerized into F-actin with a highly dynamic structure. F-actin is unstable and has the function of disaggregation. F-actin polymerization and depolymerization between the state of conversion, that is, F-actin recombination, not only to maintain cell traits and location, but also mediate intercellular connections (such as: close connection, adhesion link, fissure link). The integrity of F-actin is essential for maintaining the normal function of cells. F-actin is a major cytoskeletal contraction protein. The increase of F-actin content can increase polymerization and form stress fibers, resulting in the cell tight junction structure cannot be maintained, thus increasing the permeability of monolayer, resulting in impairment of barrier function. We showed that LPS induced actin destabilization, which led to the hyperpermeability of FLSs in turn. The present results revealed that morphologically, GE attenuated LPS-induced stress fiber formation by F-actin, which further confirmed that GE has an inhibitory effect on the LPS-induced hyperpermeability of FLSs.

Recent studies proved that the p38MAPK, Rho/ROCK pathways (Monickaraj et al., 2016; Ni et al., 2017) are involved in the regulation of microfilament assembly or contractility, thereby resulting in increased permeability. Studies found that thrombin and other stimulant can increase the F-actin polymerization to form stress fibers, and destroy the tight connection of cells, cause blood – joint capsule fluid barrier and other physiological barrier permeability increase, result in or aggravate the condition (Poli et al., 2002). Therefore, intervention of F-actin recombination may become a new target for RA treatment. Studies have shown that RhoA regulates actin polymerization/depolymerization through the Rho/ROCK pathway, causing actin recombination (Stanley et al., 2014). Activation of p38MAPK and NF-κB is an important cause of F-actin depolymerization and recombination (Yu et al., 2016). It was found that F-actin recombination and cell permeability were increased in pulmonary microvascular endothelial cells (PMVEC) induced by influenza virus, and the phosphorylation level of Ezrin/radixin/moesin (ERM) was up-regulated. ERM is the connection between the cell membrane and the actin cytoskeleton. Rho/ROCK inhibitor Y27632, p38MAPK inhibitor SB203580 can down-regulate ERM phosphorylation levels, inhibit F-actin recombination, and improve PMVEC cell permeability (Zhang et al., 2014). Thus, RhoA/p38MAPK/NF-κB/F-actin signaling pathway all contributes to hyperpermeability in cells via regulating F-actin. The current result is consistent with the previous report. The present results further demonstrated that the RhoA/p38MAPK/NF-κB/F-actin signaling pathway is activated in response to LPS and caused F-actin rearrangement, which in turn mediated hyperpermeability of FLSs.

Geniposide alleviated hyperpermeability of the FLSs *in vitro*. Some studies have reported that GE has a therapeutic effect on RA. For instance, GE downregulated expression of TNF-α, IL-2 and upregulated IL-1β, IL-4, TGF-β1 (Pan et al., 2017; Zhang et al., 2017). GE also has protective effects on LPS-induced endothelial barrier dysfunction (Xu et al., 2017). GE suppresses the release of inflammatory mediators, thus moderating the systemic inflammatory response. There have been no previous studies reporting direct effects of GE on FLSs permeability under inflammatory conditions. This study provided the first evidence to confirmed that GE directly modulated the stability of the permeability of the FLSs when exposed to the inflammation mediator and LPS. Moreover, our study confirmed that GE could regulate the inflammatory response in RA. This is GE’s new mechanism for treating RA. We found that the molecular mechanism behind GE moderation of the permeability disorder of FLSs induced by LPS is via the RohA/p38MAPK/NF-κB/F-actin signal pathway. After stimulation by LPS, the expression of RhoA, p-p38MAPK, NF-κB p -p65 and F-actin increased significantly in AA group. It demonstrated LPS activated RhoA/p38MAPK/NF-κB/F-actin signaling pathway and subsequently led to reassembly of actin, morphological changes, and increased permeability. These results suggested that GE was similar to RhoA/ROCK inhibitor Y-27632, which inhibited the intervention of RhoA in FLSs via RhoA/p38MAPK/NF-κB/F-actin signaling pathway and reduced the hyperpermeability of the FLSs, and thus playing a therapeutic role.

## Conclusion

In summary, the mechanism of GE treatment of AA may be for two aspects. On the one hand, the expressions of pro-inflammatory cytokines IL-1β and IL-17 were down-regulated and the expressions of anti-inflammatory cytokines IL-4 andTGF-β1 were up-regulated to restore the dynamic balance of pro-inflammatory cytokines and anti-inflammatory cytokines, thereby inhibiting the development of inflammation. On the other hand, GE inhibited LPS-induced RohA/p38MAPK/NF-κB/F-actin pathway in FLSs. GE attenuated LPS-induced stress fiber formation and increased permeability of FLSs via inhibition of the RohA/p38MAPK/NF-κB/F-actin pathway. These observations indicated that GE may be an effective drug for treating RA in the clinic. Finally, our data suggested that the RohA/p38MAPK/NF-κB/F-actin pathway may be a new target for the treatment of RA.

## Author Contributions

Participated in research design: RD, FL, and HW. Conducted the experiments: RD, FL, W-yW, LD, and Z-rZ. Contributed new reagents or analytic tools: RD, FL, and LD. Performed the data analysis: RD, FL, and JF. Wrote or contributed to the writing of the manuscript: RD and FL.

## Conflict of Interest Statement

The authors declare that the research was conducted in the absence of any commercial or financial relationships that could be construed as a potential conflict of interest. The reviewer YH and handling Editor declared their shared affiliation.
